# Clinical effect and biological mechanism of exercise for rheumatoid arthritis: A mini review

**DOI:** 10.3389/fimmu.2022.1089621

**Published:** 2023-01-06

**Authors:** Zongpan Li, Xue-Qiang Wang

**Affiliations:** ^1^ Department of Sport Rehabilitation, Shanghai University of Sport, Shanghai, China; ^2^ Department of Sport Rehabilitation Medicine, Shanghai Shangti Orthopaedic Hospital, Shanghai, China

**Keywords:** exercise, clinical effect, biological mechanism, rheumatoid arthritis, review

## Abstract

Rheumatoid arthritis (RA) is a common systematic, chronic inflammatory, autoimmune, and polyarticular disease, causing a range of clinical manifestations, including joint swelling, redness, pain, stiffness, fatigue, decreased quality of life, progressive disability, cardiovascular problems, and other comorbidities. Strong evidence has shown that exercise is effective for RA treatment in various clinical domains. Exercise training for relatively longer periods (e.g., ≥ 12 weeks) can decrease disease activity of RA. However, the mechanism underlying the effectiveness of exercise in reducing RA disease activity remains unclear. This review first summarizes and highlights the effectiveness of exercise in RA treatment. Then, we integrate current evidence and propose biological mechanisms responsible for the potential effects of exercise on immune cells and immunity, inflammatory response, matrix metalloproteinases, oxidative stress, and epigenetic regulation. However, a large body of evidence was obtained from the non-RA populations. Future studies are needed to further examine the proposed biological mechanisms responsible for the effectiveness of exercise in decreasing disease activity in RA populations. Such knowledge will contribute to the basic science and strengthen the scientific basis of the prescription of exercise therapy for RA in the clinical routine.

## 1 Introduction

Rheumatoid arthritis (RA) is a type of systematic, chronic inflammatory, autoimmune, and polyarticular disease that causes a range of clinical manifestations, including joint swelling, redness, pain, stiffness, fatigue, decreased quality of life, progressive disability, cardiovascular problems, and other comorbidities (1–3). RA affects 0.5%–1.0% of adults, with an annual incident rate of 5–50/100,000 person-years. The prevalence of RA in women is higher than that in men and increases with age (4–7). RA ranks 42nd among 291 conditions causing disability globally, accounting for 0.49% of the total years of living with disability (8). With the growing population and aging, the overall burden of RA, estimated by disability-adjusted life years, increased from 3.3 million in 1990 to 4.8 million in 2010 (9). Aside from the high disease burden, RA has substantial economic impact. The total average medical costs for individual RA patient ranged from US$ 5720 to US$ 5822, accounting for 8%–24% of the total medial costs, 8%–21% of physician visits, and 17%–88% of in-patient stays (10). In addition, the average number of days of absences due to RA ranges from 2.7 days/year to 30 days/year (11, 12).

Current practice involves the usage of disease-modifying drugs, and biological agents can substantially improve disease activity and minimize structural damage (13, 14). However, challenges in the current practice of RA management should be considered. Some patients remain difficult to treat or can barely achieve the targeted clinical remissions or low disease activity (15). Nonpharmacological treatments, such as exercise therapy, are promising approaches for symptom control and daily function improvement and widely used for patients with RA (16). Moreover, exercise intervention can effectively improve cardiorespiratory fitness, reduce the risk of commensurate cardiovascular disease, and decrease disease activity and severity in patients with RA (17). Thus, physical exercise therapy, as a cost-effective approach, in conjunction with drug therapy has been recommended by the European Alliance of Associations for Rheumatology (EULAR) in 2018 (18).

The clinical effect of exercise on RA has been extensively studied, and the results have been well synthesized by several reviews published from 1998 to 2022 (19–29). An expert review from Metsios et al. discussed the physiological mechanisms by which exercise alleviates inflammation, psychologic health, and cardiovascular risk in patients with RA and provided detailed description on how to incorporate exercises into RA management (30). Moreover, a systematic review from Sveaas et al. reported that high evidence supporting exercises are beneficial for reducing RA disease activity (31). However, to the best of our knowledge, the possible biological mechanisms of how exercise can decrease the RA disease activity have not been comprehensively reviewed. Such information would be essential for understanding the basic science, which can promote the administration of exercises in the routine management of RA.

## 2 Clinical effect of exercise for patients with rheumatoid arthritis

To summarize the evidence regarding the clinical effect of exercise on RA, we conducted a literature search using the keywords (exercise, physical activity, clinical effect, and rheumatoid arthritis) in PubMed and Google scholar to identify related individual studies and review articles. As there are overlaps between the individual studies and review articles and some reviews have a specific focus (e.g., a certain type of exercise or clinical domain) (24, 29, 32, 33), we chose to review the individual original studies and provide an overall summary of the clinical effect of exercise for patients with RA (**Table 1**). A total of 30 related studies published between 1985 and 2019 were identified and reviewed, of which 27 were randomized controlled trials (RCTs (34–42, 44–59, 61, 62), 2 were non-RCTs (17, 60), and 1 was before-after trial (43).

**Table 1:** Characteristics of Included Studies.

Study		RA patients			Exercise						Clinical effects (↥ improved; – no effect; ↧ worsened)									
Author, year	Design	*n*	Age ^†^	Disease status	Intervention group (IG)			Control group (CG)			DA	Swollen	Pain	Stiffness	Fatigue	QoL	P-F	CP-F	PSS/Sleep	Effective time
					Type	Frequency and Duration	Intensity	Type	Frequency and Duration	Intensity										
Lange et al. (34)	RCT	73	69.6 ± 2.5	Low–moderate DA: DAS28 < 5.1	Gym-based aerobic and resistance exercises	20 weeks x 3 sessions x 27 mins	Aerobic exercise: 70–89% HR_max_; resistance exercise: 70–80% 1 RM	Home-based exercises	20 weeks x 3 session x 27 mins	Light intensity							**↥**	**↥**		**After 20-week exercises**, aerobic capacity (VO_2_), endurance, strength (STS), dynamic balance (TUG), exercise load (LTPAI), and self-reported health (PGIC) were significantly improved (IG vs CG). **At 12-month post-exercise follow-up**, positive effect on endurance was persisted.
Siqueira et al. (35)	RCT	100	54.1 ± 6.1	Low–moderate DA and Median years with RA: 7.7–9.2	Land-based aerobic exercises	16 weeks x 3 sessions x (15–30) mins	Borg CR-10 force scale: gradually from 4–8	“Patients in the control group did not participate in any physical activities, and these patients were also instructed to continue their normal routines.”			**↥**		**↥**				**↥**			**After 8- and 16-week exercises**, DA (DAS28), pain (VAS and pain killer consumption), and functional capacity (modified HAQ) were significantly improved in water-based exercise group. Muscle strength was unchanged. The water-based exercise showed best compliance.
					Water-based aerobic exercises															
Williamson et al. (36) and Lamb et al. (37)	RCT with extended follow-up	488	62.4 ± 11.6	Median years with RA: 10 in IG and 10 in CG	Exercises (mobility, strength, and endurance) in addition to the usual care	11–16 weeks x 7 sessions x 1 set x repetitions (5–10 for mobility; 8–12 for strength)	Borg CR-10 force scale: gradually from 2–6	“Usual care (joint-protection education and functional splinting) with a maximum of three sessions of outpatient therapy were allowed, to a maximum of 1.5 h contact time.”			**–**	**↥**	**↥**				**↥**	**↥**	**–**	**After around 4-month exercises**, overall hand function (MHQ, MHQ ADL, MHQ satisfaction, MHQ pain, strength), self-efficacy, tender joint, and swollen joint were significantly improved (IG vs CG). C-reaction protein level was significantly decreased (IG vs CG). A trend showed that the physical status (SF-12 physical) was improved, with significant improvement in IG. **At 12-month post-exercise follow-up**, overall hand function (MHQ, MHQ ADL, MHQ work, strength) and dexterity were significantly improved (IG vs CG). A trend showed that the mental status (SF-12 mental) was improved, with significant improvement in IG. No significant group difference was found in DA, joint deformity, and ROM at any assessment. **At around 26-month (19**–**40 months) post-exercise follow-up**, no significant group difference in hand function scores was found (IG vs CG). However, compared to the baseline, the clinical effects were significantly persisted in IG, but not in CG.
Lourenzi et al. (38)	RCT	60	51.7 ± 7.9	Median years with RA: 9.1 in IG and 11.3 in CG	Progressive resistance strength exercises	12 weeks x 2 sessions x (50–60) mins	50–70% 1 RM	“Same to the IG, the patients in the CG kept the conventional drug treatment during all the study time.”			**–**		**↥**			**↥**	**↥**			**After 6- and 12-week exercises** and **24-month post-exercise follow-up**, physical function (HAQ), quality of life (SF-36 in functional capacity and pain domains), muscle strength, and patients’ satisfaction with treatment (Likert scale) were significantly improved (IG vs CG). No group difference in DA (DAS28) and pain was found. But pain was significantly improved in IG and CG.
Manning et al. (39)	RCT	108	55.1 ± 15.5	Median years with RA: 1.7 (all ≤ 5)	Education, self-management, and upper extremity exercise training	2 weeks x 2 sessions supervised group training + 12 weeks x 7 sessions home exercises x duration (NR)	13–17/20 RPE	“Patients in CG received usual care continued to be managed by their medical team.”			**↥**		**↥**			**–**	**↥**			**After 12-week exercises**, physical disability (30-item DASH), physical function (GAT), strength, self-efficacy (ASES pain and symptoms subscales), DA (DAS28), and self-reported pain (VAS) were significantly improved (IG vs CG). **At 36-month post-exercise follow-up**, effects on self-efficacy (ASES pain) and self-reported pain were persisted.
Durcan et al. (40)	RCT	78	60.0 ± 10.0	Mean years with RA: 16 in IG and 11 in CG	Cardiovascular exercise	12 weeks x 5 sessions x (30–60) mins	Light to moderate	“Patients in CG received standard care and were given verbal and written instruction regarding the benefits and importance of exercise in RA.”					**↥**	**↥**	**↥**		**↥**		**↥**	**After 12-week exercises**, physical function (HAQ), pain (VAS), stiffness (VAS), subjective sleep quality (PSQI), and fatigue (FFS) were significantly improved (IG vs CG).
					Resistance training	12 weeks x 2–3 sessions x 2 sets x (30–60) mins	50–70% 1 RM													
					Flexibility and neuromotor exercises	12 weeks x 2–3 sessions x 2–4 sets x 30s	Stretch to the point of tightness													
Jahanbin et al. (41)	RCT	65	48.8 ± 9.8	Mean duration of RA for most cases > 5 years	Aerobic, isometric, and isotonic exercises	8 weeks x 2 sessions x 45 mins	NR	“The patients in CG only received a training complied with the routine program of the clinic.”					**↥**				**↥**		**↥**	**After 8-week exercises**, healthy status (AIMS2-SF: physical health, symptom, psychological, social interaction, and function domains), and pain (VAS) were significantly improved (IG vs CG).
da Silva et al. (42)	RCT	102	58.2 ± 8.3	Mean years with RA: 9.7 in IG and 9.6 in CG	Sensorimotor exercises	16 weeks x 2 sessions x (30–50) mins	NR	“The control group was only submitted to the clinical drug treatment with Methotrexate, Leflunomide and/or Prednisone (5 mg).”					**↥**			**↥**	**↥**			**After 8-week exercises**, the quality of life (SF-36), and functional capacity (HAQ and TUG) were significantly improved (IG vs CG). In addition, the functional variables (HAQ and TUandGT), pain (VAS) and Balance and Gait Scales (BBS and TINETTI) were significantly improved in IG.
Stavropoulos –Kalinoglou et al., (17)	NRCT	36	53.9 ± 9.9	Median years with RA: 5.5 in IG and 7.0 in CG	Individualized aerobic and resistance exercises	24 weeks x 3 sessions x (50–60) mins	Aerobic exercise: 70% VO_2 max_; resistance exercise: 70% 1 RM	“Patients in CG received advice on exercise benefits and lifestyle changes”			**↥**				**↥**		**↥**	**↥**		**After 12- and 24-week exercises**, physical disability (HAQ), DA (DAS28), cardiorespiratory fitness and fatigue (MAF) were significantly improved (IG vs CG).
Karatepe et al. (43)	BAT	28	52.9 ± 8.6	Low to moderate DA and ARA functional class I–II	Home-based strengthening and ROM exercises	4 weeks x 5 sessions x 2 sets x (10–15) repetitions	NR	–			**–**					**↥**	**↥**			**After 4-week exercises**, functional status (HAQ) and health-related quality of life (RAQoL) were improved (IG vs CG). DA (DAS28) was unchanged. **At 12-month post-exercise follow-up**, such effects were persisted. DA was unchanged.
Baillet et al. (44)	RCT	48	53.9 ± 10.8	ACR I–II	Dynamic exercise program	4 weeks x 5 sessions x 105 mins	60–80% HR_max_	Educational films with hydrotherapy, and physical exercises	3 days x 45 mins	To prevent muscle atrophy	**–**					**↥**	**↥**	**↥**		**After 4-week exercises**, functional status (HAQ), quality of life (NHP), and aerobic fitness were improved (IG vs CG). DA (DAS28) was unchanged. **At 6- and 12-month post-exercise follow-ups**, such effects were not persisted.
Flint-Wagner et al. (45)	RCT	24	51.0 ± 12.8	Mean ± SD with RA: 14.0 ± 10.2	High-intensity strength training	16 weeks x 3 sessions x 75 mins	70–85% 1 RM	“Patients in CG continued with standard of care, as overseen by their rheumatologists”					**↥**				**↥**			**After 16-week exercises**, the physical functions (HAQ, 50-foot walk test, and grip strength) and pain were improved (IG vs CG).
Hsies et al., (46)	RCT	30	52.7 ± 10.2	ACR II–III	Supervised aerobic exercises	8 weeks x 3 sessions x 60 mins	50–80% VO_2 peak_	Home-based aerobic exercises	8 weeks x 3 session x 60 mins	50–80% VO_2 peak_	**–**	**–**	**↥**				**–**	**↥**		**After 8-week exercises**, aerobic capacity (VO_2_, work, MET, O_2_ pulse) was improved (IG vs CG), and the supervised exercises was superior to the home-based exercises. Pain was evenly reduced in IG and CG. The swollen joints, functional status (HAQ), and DA (ESR) were unchanged.
Lemmey et al. (47)	RCT	28	58.3 ± 10.1	Mean years with RA: 6.2 in IG and 10.4 in CG	High-intensity progressive resistance training	24 weeks x 2 sessions x 3 sets x 8 repetitions	80% 1 RM	“Control subjects were asked to perform these ROM exercises twice weekly at home.”			**–**						**↥**			**After 24-week exercises**, the physical functions (30-second arm curl test, chair test, 50-foot walk test, and knee extensor strength) were improved (IG vs CG). IGF level was increased (IG vs CG). DA (DAS28 and ESP) was unchanged.
Neuberger et al. (48)	RCT	220	Range: 40–70	Median years with RA 8.0 (range 0.5–50)	Class exercises or home exercises	12 weeks x 3 sessions x 60 mins	60–80% HR_max_	“Participants in the control group were asked to keep exercise levels at baseline amounts.”			**–**		**↥**		**↥**		**↥**		**↥**	**After 6- and 12-week exercises**, physical function (walk time and grip strength), pain (McGill pain), fatigue (MAF), and depression (CES-D and POMS depression) were improved (IG vs CG). DA (ESR) were unchanged.
Berg et al., (49)	RCT	160	49.7 ± 13.4	Median years with RA: 7.6 in IG and 5.5 in CG	Individualized and internet-based physical activity	48 weeks x 5 sessions x 3 sets x 10 repetitions	60–80% HR_max_ and 4–5/10 RPE	Internet-based recommender physical activity	48 weeks x 5 session x 30 mins	Low to moderate	**–**					**↥**	**↥**		**–**	**After 6-, 9-, and 12-month exercises**, the proportion of patients who were physically active at moderate/vigorous intensity level were significantly higher (IG vs CG). No significant difference was found for mental summary scales and DA (DAS28) between the groups. **After 9-month exercises**, quality of life (RAQoL) was significantly greater (IG vs CG).
Melikoglu et al. (50)	RCT	40	48.4 ± 9.1	ACR I–II	Dynamic or ROM exercises	2 weeks x 5 sessions x 20 mins	60% HR_max_	Healthy participants with dynamic exercises	2 weeks x 5 session x 20 mins	60% HR_max_			**↥**	**–**						**After 1- and 2-week exercises**, pain (VAS and RAI) was significantly improved by the dynamic exercises. IGF-1 level was significantly increased by dynamic exercises, while that was decreased in ROM exercise and control groups. Morning stiffness was unchanged.
Bilberg et al. (51)	RCT	46	Range: 21–65	Median years with RA: 2.6 in IG and 2.9 in CG	Pool exercise therapy	12 weeks x 2 sessions x 45 mins	Moderate aerobic intensity	“Patients in the control group continued their daily activities, including a home exercise program introduced on admission to the clinic.”									**↥**	**–**		**After 3-month exercises**, muscular functions (endurance and strength) were significantly improved (IG vs CG). No significant improvement in aerobic capacity. **At 6-month post-exercise follow-up**, such effects were persisted.
Hakkinen et al., (52) and (53)	RCT with extended follow-up	62	49.0 ± 10.5	Median years with RA: 0.8 in IG and 0.7 in CG	Strength training	96 weeks x 2 sessions x 2 sets x (8–12) repetitions	50–70% 1 RM	ROM and stretching exercises	96 weeks x 2 session	NR	**↥**						**↥**			**After 2-year exercises**, muscle strength, DA (DAS28 and ESR), and physical function (HAQ) were significantly improved (IG vs CG). **At 5-year post-exercise follow-up**, such effects were persisted.
De Jong et al. (54)	RCT	300	Range: 20–70	Median years with RA: 7.5 in IG and 5.0 in CG	Combined exercises	96 weeks x 2 sessions x 75 mins	70–90% HR_max_	“Patients assigned to the CG were treated by a physical therapist only if this was regarded as necessary by their attending physician.”			**–**						**↥**	**↥**	**↥**	**After 1-year exercises**, physical function (HAQ and MACTAR questionnaire score), muscle strength, and emotional status (HADS) were significantly improved (IG vs CG). DA (DAS4) was unchanged. **After 2-year exercises**, physical function (HAQ and MACTAR questionnaire score and HAQ), aerobic fitness, muscle strength, and emotional status were significantly improved (IG vs CG). DA was unchanged.
Bearne et al. (55)	RCT	103	Range: 30–82	Mean years with RA 11.0 (range 2–40)	Progressive, individually prescribed exercises	5 weeks x 2 sessions x (30–45) mins	“The patient was encouraged to work as hard as they could”	“Patients randomized to the control group continued their normal activities”				**–**	**–**	**–**			**↥**			**After 5-week exercises**, quadriceps function (strength and activation) was improved (IG vs CG). Pain, stiffness, and swollen joint were unchanged. **At 6-month post-exercise follow-up**, the improvement on quadriceps function was maintained.
Buljina et al. (56)	RCT	100	Range: 20–70	Mean years with RA 5.2 (range 0.5–22)	Physical and exercise therapy	3 weeks x 7 sessions x (20–30) mins	NR	“At the time of the investigation, patients in CG were not receiving any physical or exercise therapy”					**↥**			**↥**	**↥**			**After 3-week exercises**, the hand function (ROM and strength), ADL, and pain were significantly improved (IG vs CG).
Van den Ende et al. (57)	RCT	64	60.0 ± 13.0	Median years with RA: 8.0 in IG and 7.0 in CG	Extra intensive exercises, in addition to the usual conservative exercises	24 weeks x 5 sessions x 3 sets x 5 repetitions	70% MVC and 60% HR_max_	Usual conservative exercises (ROM and isometric)	24 weeks x 5 session x 3 sets x 5 repetitions	NR	**↥**	**–**	**↥**	**–**			**↥**			**After 3-, 6-, 12-, and 24-week exercises**, DA (ESR) showed a gradual decline in IG and CG, with greater improvement in IG. Pain (VAS) was improved significantly more in CG than that in IG after first **3-week exercises**, while during the later assessments the improvement was the same in both groups. Joint mobility in IG was improved after **12-, and 24-week exercises.** Muscle strength was significantly and gradually improved (IG vs CG). Functional ability (HAQ) was improved over time in both groups. IG showed a slight increase in swollen joints after first **3-week exercises**, followed by a decline thereafter. No significant difference was found in the number of swollen joints at any evaluation.
Westby et al. (58)	RCT	23	53.8 ± 10.8	ACR I–II	Aerobic dance and strengthening exercises	48 weeks x 3 sessions x (45–60) mins	60–75% HR_max_	“Patients continued with regular physical activities and therapy as needed and were also given written materials on osteoporosis and a pamphlet on exercise and arthritis.”			**↥**						**↥**	**↥**		**After 1-year exercises**, active joints were reduced, and ESR was decreased in IG, with a slight increase in the CG. There was no significant difference in DA (IG vs CG). The HAQ, fitness level, Caltrac activity level were improved in IG, but not in CG.
Van den Ende et al. (59)	RCT	100	Range: 20–70	Median years with RA: 11.5 in IG and 8.4–11.2 in CG	High intensity group exercises	12 weeks x 3 sessions x 60 mins	70–85% HR_max_	Low intensity group exercises	12 weeks x 2 session x 60 mins	Low	**–**	**↥**	**–**	**–**			**↥**	**↥**		**After 12-week exercises**, aerobic capacity (VO_2 peak_), joint mobility (EPM-ROM score), muscle strength, swollen joints and observed functional ability (walk test) were significantly improved, while functional ability (HAQ and Dutch AIMS), and DA (ESR, VAS pain, and morning stiffness) showed no significant change (IG vs CG). **At 24-week post-exercise follow-up**, the improvements muscle strength and observed functional ability were maintained in IG. No significant difference was found in measured functional ability (HAQ and Dutch AIMS) or DA.
								Low intensity individual exercises	12 weeks x 2 session x 60 mins	Low										
								Home exercise	12 weeks x 2 session x 15 mins	NR										
Noreau et al. (60)	NRCT	29	49.3 ± 12.4	Median years with RA: 8.1 in IG and 11.0 in CG	Dance-based aerobic exercise	12 weeks x 2 sessions x (25–45) mins	50–70% HR_max_	“Patients not participating the exercise program served as the controls.”			**–**		**–**				**↥**	**↥**	**↥**	**After 12-week exercises**, cardiorespiratory fitness (VO_2 max_ and maximum workload), psychological (AIMS and POMS), functional ability (walk test), and muscle strength (hamstrings) were significantly improved in IG, but not in CG. A trend showed that pain (painful joints and AIMS) was improved in IG. DA and physical function measured by AIMS subscales were unchanged in both groups. **At 9-month post-exercise follow-up**, such effects were not persisted.
Lyngberg et al. (61)	RCT	24	66.5 ± 9.1	Median years with RA: 10.5 in IG and 7.0 in CG	Combined exercises with low-dose steroids	12 weeks x 2 sessions x 45 mins	50–70% HR_max_	“Patients received low-dose steroids and were allowed to continue with their usual physical activities.”			**–**	**–**	**–**	**–**			**↥**	**–**		**After 12-week exercises**, functional ability (step-climbing and heel lifts) and muscle strength were significantly improved (IG vs CG). Aerobic ability (VO_2 max_), Pain, swollen joints, morning stiffness, DA (DAS28) was unchanged.
Harkcom et al. (62)	RCT	20	52 ± 12	Median years with RA: 5.6–12.2 in IG and 8.8 in CG	Aerobic exercises	12 weeks x 3 sessions x 15-35 mins	70% HR_max_	“Control subjects continued their routine daily activities without knowledge of any intervention.”				**↥**	**↥**					**↥**		**After 12-week exercises**, aerobic capacity, joint counts on pain and swollen, and exercise tolerance were significantly improved (IG vs CG).
**Summary in the clinical effects of exercise for RA patients (for each clinical domain: number of studies reported to be improved/total studies reported):**											**DA**	**Swollen**	**Pain**	**Stiffness**	**Fatigue**	**QoL**	**P-F**	**CP-F**	**PSS/Sleep**	
											**7/20**	**4/8**	**15/19**	**1/6**	**3/3**	**6/7**	**27/28**	**11/13**	**5/8**

^†^ Mean ± SD (years), unless other indicates; ACR, American college of rheumatology classification of global functional status; DA, disease activity; QoL, quality of life; P-F, physical function; CP-F, cardiopulmonary function; PSS, psychosocial function; RCT, randomized controlled trail; NRCT, non-randomized controlled trail; BAT, before-after trail; RA, rheumatoid arthritis; DAS, disease activity score; ARA, American rheumatology association; ROM, range of motion; RPE, Borg Rating of Perceived Exertion; 1RM, one repetition maximum; HR_max_, maximum heart rate; VO_2 peak_, peak oxygen uptake; VO_2 max_, maximum oxygen uptake; HAQ, health assessment questionnaire; VO_2_, oxygen consumption; MET, metabolic equivalent; ESR, erythrocyte sedimentation rate; MAF, multidimensional assessment of fatigue; CES-D, center for epidemiological studies depressions scale; POMS, profile of mood states form; TUG, timed up and go; STS, sit to stand; LTPAI, the leisure time physical activity instrument; PGIC, the patient’s global impression of change; RAQoL, rheumatoid arthritis quality of life scale; MACTAR, McMaster toronto arthritis patient preference disability questionnaire; RAI, Ritchie articular index; IGF-1; insulin-like growth factor-1; HADS; hospital anxiety and depression scale; VAS, visual analogue scale; Dutch AIMS, Dutch arthritis impact measurements scales; DASH, Disabilities of the Arm, Shoulder, and Hand; GAT, grip ability test; ASES, arthritis self-efficacy scale; FFS, fatigue severity scale; PSQI, Pittsburgh sleep quality index; BBS, berg balance scale; TINETTI, Tinetti test; ADL, activities of daily living; NR, not reported. The time points for post-exercise assessment are in bold.

Exercise interventions are effective in improving physical ability (17, 34–45, 47–49, 51–61), alleviating pain (35–42, 45, 46, 48, 50, 56, 57, 62), and improving aerobic function (17, 34, 36, 37, 44, 46, 54, 58–60, 62) in patients with RA. In addition, exercise can be effective in improving quality of life (38, 42–44, 49, 56), mental health or sleep status (40, 41, 48, 54, 60), and fatigue (17, 40, 48) and does not aggravate disease activity (17, 35–39, 43, 44, 46–49, 52–54, 57–61) or severity of some conditions, including swollen joints (36, 37, 46, 55, 57, 59, 61, 62) and joint stiffness (40, 50, 55, 57, 59, 61). Exercise training for relatively longer periods can decrease disease activity or clinical severity (i.e., 12–96 weeks) (17, 35–37, 39, 40, 52, 53, 57, 58). The duration of an effective exercise ranges from 2 weeks to 96 weeks, suggesting even short-term exercises can be clinically beneficial for patients with RA. For safety considerations, most exercise protocols utilize moderate intensities for strengthening (i.e., 50%–70% one repetition maximum: 1 RM) and aerobic training (i.e., 50%–70% maximum heart rate: HR_max_ or peak oxygen uptake: VO_2 max_). However, several studies have shown that high-intensity strengthening (i.e., 1 RM ≥ 70%) and aerobic (i.e., HR_max_ or VO_2 max_ ≥ 70%) exercises are effective for patients with RA, and no exercise-related adverse events have been reported (17, 34, 45, 47, 54, 59, 62). Hence, exercises with moderate-to-high intensities are clinically effective and safe for patients with RA. Exercise therapies seem effective and safe for patients with various stages of RA (i.e., duration 0.5–50 years). More importantly, exercises can effectively promote clinical remission in patients with relatively early RA stages (i.e., duration ≤ 5 years (39, 51–53). Hence, exercise therapies are safe and cost-effective approaches and provide a “window of opportunity” for the early management of RA in first-line treatment (63).

In summary, exercise is effective in alleviating pain, improving physical ability, aerobic function, quality of life, mental health, and sleep status and reducing fatigue in patients with RA. Exercise training for relatively longer periods is effective in reducing disease activity.

## 3 Biological mechanism of exercise for rheumatoid arthritis

**Figure 1:**
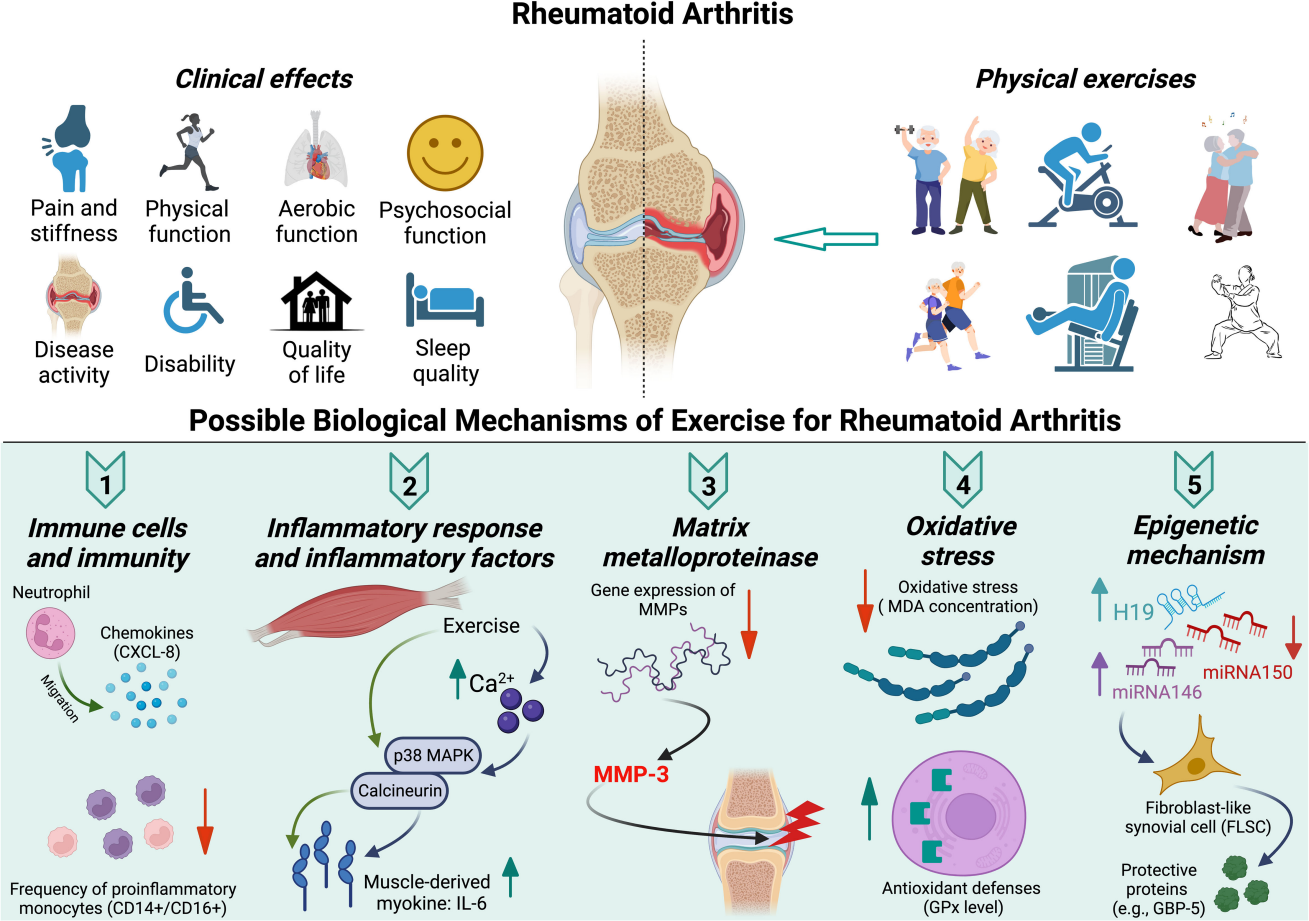
Clinical effects and possible biological mechanisms of exercise for rheumatoid arthritis.

Exercise enables a range of biological responses, including the immune systems (64), inflammation (65), matrix metalloproteinase (MMP) (66), oxidative stress (67), and epigenetic adaptation (68). Exercise may reduce the RA disease activity from the following biological aspects: immune cells and immunity, inflammatory response and inflammatory factors, MMP, oxidative stress, and epigenetic expression (**Figure 1**).

### 3.1 Immune cells and immunity

RA is known as a chronic autoimmune disease, with a maladaptive tissue repair process elicited by multiple types of immune cells and malfunction of signaling networks (69). Exercise can profoundly affect the immune system, and causes regulation of immune functions (64), which may reduce the RA disease activity. A single bout of brief dynamic exercise (several minutes) causes an increase in the leukocyte count to 2 to 3 times, whereas prolonged exercise (0.5–3 hours) may increase the count of leukocytes up to fivefold (70, 71). Although the increase of leukocyte count is a common indicator of infection/inflammation, the increase can be returned to the pre-exercise level within 6–24 hours after cessation of the exercise (64). Particularly after endurance exercises, the lymphocyte count in blood would be falling 30–50% below pre-exercise level, reaching the clinically low level (i.e., < 1.0 x 10^9^/L) (64, 70, 71). In addition, the neutrophils and lymphocytes can be predominantly mobilized by exercise (70, 71), and the exercise-mobilized cells have increased effector/cytotoxic functions (64).

Growing evidence has shown that exercise and habitual physical activity enhance the immune function of adaptive and innate cells in healthy adults (70, 72). Several studies have examined the effect of exercise on immune response in patients with RA. One study investigated the effect of an 8-week bicycle exercise on immune response in patients with RA and observed temporary increase in lymphoproliferative response during the acute phase of the exercise and no significant changes in the levels of blood mononuclear cell populations in the post-exercise resting state (73). Another study assessed immune responses to exercise in patients with RA and found no changes in lymphocyte proliferation and natural killer cells, suggesting that exercise does not enhance primary cell functions in RA (74). A more recent study that examined the effects of exercise training on immune function in stable patients with RA showed that the neutrophil migration toward chemokines (CXCL-8) was promoted and the frequency of proinflammatory monocytes (CD14+/CD16+) in the circulation was reduced after exercise (75). As RA is characterized by the dysfunctions of peripheral blood neutrophil migration and increased frequency of proinflammatory monocytes (69, 76), exercise may reduce RA disease activity by improving innate immune functions from the two above-mentioned aspects.

### 3.2 Inflammatory response and inflammatory factors

Once immune cells detect an infection or tissue injury, inflammation is triggered by the innate immune system (69). Certain inflammatory cytokines (e.g., interleukin: IL-6; and tumor necrosis factor alpha: TNF-α) are related to the pathogenesis and progression of RA (77). Clinically, inflammatory markers (i.e., c-reactive protein: CRP) and erythrocyte sedimentation rate (ESR) are routinely used in detecting and monitoring inflammation. In addition, ESR has been commonly used as a component in the calculation of disease activity (e.g., DA28-ESR) (78, 79). Evidence from RCTs has shown that exercise can effectively decrease inflammation/disease activity (i.e., exercise-induced decrease in ERS) in patients with RA (52, 57, 58). However, the biological mechanism of such effects remains unclear.

Although IL-6 is commonly viewed as a proinflammatory cytokine, accumulating evidence has shown that the muscle-derived IL-6, known as a type of myokine, has anti-inflammatory functions (80). Different from the signaling pathway of the expression of IL-6 in macrophages during sepsis (i.e., dependent upon the activation of NF-κB), the contraction of the skeletal muscle causes an increased cytosolic Ca^2+^ and increased activation of p38 MAPK/calcineurin, which facilitates the production of IL-6 but not of TNF (81). IL-6 is the first cytokine released into the blood during exercise (82). In general, inflammatory cytokine level decreases within a few hours after an exercise (83, 84). However, evidence of the acute effect of exercise on inflammatory response in patients with RA is inconsistent (85). Two observational studies examined the acute effect of a single-session exercise on inflammatory cytokine (e.g., IL-6) in patients with RA; one of the studies found no significant change in IL-6 (86), whereas the other observed IL-6 level sharply increased in the first one hour, then gradually decreased, and returned to pre-exercise level in 24 hours (87). Future studies are needed to clarify the role of muscle-derived IL-6 in the inflammatory response during exercise, and its potential anti-inflammatory function in RA populations.

### 3.3 Matrix metalloproteinase

Matrix metalloproteinase (MMP) constitutes a large group of zinc-dependent proteases that degrade the components of the extracellular matrix, including collagen, gelatin, casein, and elastin. Exercise may regulate MMP level by affecting their tissue inhibitor of metalloproteinase (TIMPs), transforming growth factor-β (TGF- β), or RNA expression of MMPs (88–90). Evidence shows that physical exercise training is effective in reducing MMP level in healthy men (91), sedentary women (92), individuals with metabolic syndrome (93), patients with coronary artery disease (94), patients with diabetes (95, 96), patients with multiple sclerosis (97), and female patients with postmenopausal osteoporosis (98). Overall, physical exercise can effectively reduce MMP level in different populations.

However, evidence regarding the effectiveness of exercise on MMP reduction in patients with RA is limited. Wang et al. obtained knee synovial tissues from patients with RA who underwent total knee replacement to examine whether mechanical stretching regulates MMP secretion; they found that mechanical stretching induced significant reduction in the messenger RNA expression levels of MMP-1 and MMP-13 (88). To the best of our knowledge, no *in vivo* study has explored the effectiveness of exercise in reducing MMP level in patients with RA. MMP-3 is produced in the joints, and it aggravates inflammation by activating a range of pro-MMPs and cleaving extracellular matrix components (99). Elevated serum MMP-3 level is positively associated with inflammatory mediators and the disease activity of RA (100–103) and is a crucial outcome for early RA (101–103). Thus, MMP-3 has been regarded as a reliable marker for disease activity, predictability of disease outcome, radiological monitoring, and therapeutic response for RA (104). Hence, it is important for future studies to examine the exercise-induced change in MMP-3 in RA patients and its potential role in explaining the biological mechanism of exercise-induced reduction in RA disease activity.

### 3.4 Oxidative stress

In RA, particularly at the early stage, oxidative stress may initiate and perpetuate the local and systemic inflammation process (105). Oxidative stress has detrimental effects on the structures and functions of cellular proteins and proteoglycans, *via* different processes (i.e., oxidation and nitrosylation) (106). The accumulated oxidized cellular components and damaged products may aggravate the synovial inflammation; and the damaged contents caused by oxidative stress can also be released into the extracellular spaces and increase cellular death (106).

Oxidative stress is characterized as elevated intracellular levels of reactive oxygen species (ROS), which can be indirectly measured by lipid peroxidation (e.g., malondialdehyde: MDA), protein oxidation or nitration (e.g., protein carbonyl), or DNA/RNA damage (e.g., 8-hydroxydeoxyguanosine: 8-oxo-dG) (107). Exercise-induced metabolic challenges result in elevated generation of ROS, and such exercise-related changes in the redox milieu are modulated by several factors of mitochondrial biogenesis (e.g., PGC-1α, mitogen-activated protein kinase, and SIRT1) (108). Evidence from animal and human studies suggests that exercise can effectively reduce oxidative stress and improve antioxidant defense. Findings from animal studies have shown that high-intensity aerobic exercise can effectively reduce oxidative stress (reduces MDA concentration) and improve antioxidant defenses (increases GPx) in rats (109, 110). Similar results have been obtained by studies on the effectiveness of physical exercise on oxidative stress (reduced rate and concentration of MDA; decreased 8-oxo-dG) and antioxidant defense system (increased GPx) in humans with or without diseases (111–114).

However, inconsistent results can be seen in RA populations. Wadley et al. reported that a single bout of moderate-intensity exercise increased the oxidative stress (increased protein carbonyls and nitric oxide metabolites) in RA patients, while the following 3-month exercise significantly decreased the RA disease activity, but without significant change in oxidative stress (115). In contrast, Tuna et al. found that the 30-minute aerobic exercise caused a significant reduction in MDA concentration immediately and 24 hours after the exercise in the RA group (116). It is important for future studies to further examine the effect of exercise on oxidative stress and antioxidant defenses in RA populations, and the related biological and molecular mechanisms of exercise, oxidative stress, and the resultant reduction in RA disease activity.

### 3.5 Epigenetic mechanism

A range of aberrantly expressed noncoding RNAs (miRNA, lncRNA, and circRNA) were observed in RA. For instance, the expression levels of miRNA146a/b and H19 (lncRNA) are upregulated (117–119), but the miRNA-150-5p expression is downregulated in RA (120). Regular exercise induces genome-wide epigenetic modifications in skeletal muscles and adipose tissues in the human body, which are linked with altered expression of mRNA (121, 122). Such exercise-induced alteration in DNA methylation and mRNA expression are believed to improve the metabolic phenotypes and decrease the risk of disease (68).

Evidence has shown the effectiveness of exercise in the regulation of noncoding RNAs (123–125). Four studies reported that physical exercise downgraded the expression of miRNA146 in trained males (126), active males (127), amateur basketball players (128), and patients with chronic kidney diseases (129). Findings from an animal study suggested that a 4-week moderate endurance exercise reduced the expression of H19 in the hearts of rats with myocardial infarction (123). Van Craenenbroeck et al. reported that miRNA150 expression was upregulated after 10 minutes of endurance aerobic exercise in people with chronic kidney diseases (129). Despite these findings from non-RA populations, the effectiveness of exercise in regulating noncoding RNA expression in patients with RA remains unexplored.

Noncoding RNAs, including microRNAs (miRNAs), long noncoding RNAs (lncRNAs), and circular RNAs (circRNAs), play crucial roles in the regulation of inflammation, autoimmunity, and activation, differentiation, and polarization of immune cells (130). Epigenetic disorders can activate rheumatoid arthritis synovial fibroblasts (RASFs) (131), which promote inflammation and joint destruction in RA (132). In non-inflammatory joints of healthy individuals, normal SFs are differentiated from mesenchymal stromal/stem cells under normal genetic regulation. However, aberrant epigenetic alterations would promote the activation of SFs in inflammatory joints of patients with RA (131). A recent study from Haque et al. reported that in response to cytokine stimulation, the guanylate binding protein 5 (GBP-5) from RASFs is a potential target to restore cellular homeostasis, inflammation, and tissue destruction in RA (133).

In summary, future studies are needed to examine the effect of exercise on noncoding RNA expression in RA populations. It is also important to explore the potential role of epigenetic regulation in the biological mechanism of exercise for the reduction of RA disease activity.

## 4 Conclusions

The clinical effectiveness of exercise for RA treatment has been extensively studied. Substantial evidence has shown that exercise therapies of different types, durations, and intensities can be clinically effective for RA. More importantly, exercise for relatively longer periods can decrease the RA disease activity. This review discusses the possible biological mechanism of exercise for reducing RA disease activity from the following aspects: 1) immunity; 2) inflammatory response; 3) MMP; 4) oxidative stress; and 5) epigenetic mechanism. However, the related evidence is mainly based on evidence from non-RA populations. This may highlight the urgency for future studies to further examine the proposed biological mechanisms in RA populations.

## 5 Future directions

Based on current evidence, the following directions regarding the exercise for reducing RA disease activity are proposed for further investigation in RA populations: 1) exercise may cause neutrophil migration toward chemokines (e.g., CXCL-8) and reduce the frequency of proinflammatory monocytes (e.g., CD14+/CD16+) in the circulation; 2) exercise may promote the production of muscle-derived myokine (IL-6), which may have anti-inflammatory functions; 3) exercise may induce reduction in the messenger RNA expression of MMPs(e.g., MMP-3); 4) exercise may reduce oxidative stress (reducesMDA concentration) and improve antioxidant defenses(increases GPx level); and 5) exercise may downregulate the expression of miRNA146 and H19 but upregulate miRNA150expression, which may relate to the production of protectiveproteins (e.g., GBP-5) for decreasing RA disease activity.

## Author contributions

ZL and X-QW carried out the literature search, reviewed all the included articles, and drafted and edited the manuscript. All authors contributed to the article and approved the submitted version.
